# Automated brain volumetric measures with AccuBrain: version comparison in accuracy, reproducibility and application for diagnosis

**DOI:** 10.1186/s12880-022-00841-2

**Published:** 2022-07-04

**Authors:** Lei Zhao, Yishan Luo, Vincent Mok, Lin Shi

**Affiliations:** 1 BrainNow Research Institute, Shenzhen, Guangdong Province China; 2 Department of Medicine and Therapeutics, The Chinese University of Hong Kong, Shatin, Hong Kong SAR China; 3 Therese Pei Fong Chow Research Centre for Prevention of Dementia, The Chinese University of Hong Kong, Shatin, Hong Kong SAR China; 4 Chow Yuk Ho Technology Centre for Innovative Medicine, The Chinese University of Hong Kong, Shatin, Hong Kong SAR China; 5 Lui Che Woo Institute of Innovative Medicine, The Chinese University of Hong Kong, Shatin, Hong Kong SAR China; 6 Department of Imaging and Interventional Radiology, The Chinese University of Hong Kong, Shatin, Hong Kong SAR China

**Keywords:** AccuBrain, Version comparison, Automated brain volumetry, Accuracy, Reproducibility

## Abstract

**Background:**

Automated brain volumetry has been widely used to assess brain volumetric changes that may indicate clinical states and progression. Among the tools that implement automated brain volumetry, AccuBrain has been validated for its accuracy, reliability and clinical applications for the older version (IV1.2). Here, we aim to investigate the performance of an updated version (IV2.0) of AccuBrain for future use from several aspects.

**Methods:**

Public datasets with 3D T1-weighted scans were included for version comparisons, each with Alzheimer’s disease (AD) patients and normal control (NC) subjects that were matched in age and gender. For the comparisons of the brain volumetric measures quantified from the same scans, we investigated the difference of hippocampal segmentation accuracy (using Dice similarity coefficient [DSC] as the major measurement). As AccuBrain generates a composite index (AD resemblance atrophy index, AD-RAI) that indicates similarity with AD-like brain atrophy pattern, we also compared the two versions for the diagnostic accuracy of AD versus NC with AD-RAI. Also, we examined the intra-scanner reproducibility of the two versions for the scans acquired with short-intervals using intraclass correlation coefficient.

**Results:**

AccuBrain IV2.0 presented significantly higher accuracy of hippocampal segmentation (DSC: 0.91 vs. 0.89, *p* < 0.001) and diagnostic accuracy of AD (AUC: 0.977 vs. 0.921, *p* < 0.001) than IV1.2. The results of intra-scanner reproducibility did not favor one version over the other.

**Conclusions:**

AccuBrain IV2.0 presented better segmentation accuracy and diagnostic accuracy of AD, and similar intra-scanner reproducibility compared with IV1.2. Both versions should be feasible for use due to the small magnitude of differences.

## Introduction

Magnetic resonance imaging (MRI)-based brain volumetry has been increasingly used in the clinical settings to assess brain volumetric changes for a wide range of neurological diseases. Brain volumetric measures have been shown to be valid biomarkers of clinical state and progression by offering high reliability and robust inferences on the underlying disease-related mechanisms [1]. Automated brain volumetry with software packages of brain segmentations has been widely applied due to its superiority in efficiency and reproducibility over manual segmentations.

Among the available tools for automated brain volumetry, AccuBrain (https://www.brainnow.net/about-accubrain), as a commercial software package (cloud-based commercial system) cleared with FDA (510(k) K202847) and CE mark that performs brain structure and tissue segmentation and quantification in a fully automatic mode, has shown better accuracy (e.g., hippocampal segmentation), efficiency and inter-scanner reproducibility than the widely-used free package FreeSurfer [2, 3]. The brain volumetric measures derived from AccuBrain have also been used to identify brain structural changes in different neurological diseases (e.g., stroke [4], small vessel disease [5] and temporal lobe epilepsy (TLE) [6]), to evaluate postoperative outcomes (e.g., Cushing’s disease [7] and TLE with hippocampal sclerosis [8]), and to differentiate different dementias (e.g., vascular dementia from Alzheimer’s disease (AD) [9] and frontotemporal dementia from AD [10]). Specifically, AccuBrain also generates a composite index (i.e., AD resemblance atrophy index, AD-RAI) that assesses the similarity of brain atrophy pattern with AD based on the support vector machine model derived from an in-house training database [11], which has shown high consistency with clinical diagnosis and biological diagnosis of AD [12, 13].

Recently, AccuBrain underwent a substantial revision update (version 2.0—AccuBrain IV2.0), including an update in segmentation algorithms. In this study, we aimed to compare the recent version (IV2.0) with the previous version (IV1.2) in (1) segmentation accuracy, (2) diagnostic accuracy of AD with AD-RAI, and (3) intra-scanner reproducibility of brain volumetric measures, to provide suggestions of future use of AccuBrain in clinical applications.

## Methods

### Sources of data

#### ADNI

Data used in this work included subjects from the Alzheimer’s Disease Neuroimaging Initiative phase 1 (ADNI-1) and phase 2 (ADNI-2) who had baseline diagnostic information and MRI scanning (https://adni.loni.usc.edu/). The MRI scans were acquired from 1.5T scanners (magnetization-prepared rapid gradient echo [MPRAGE], for ADNI-1) and 3T scanners (MPRAGE or inversion recovery prepared fast spoiled gradient recalled [IR-FSPGR] for ADNI-2), with variable resolution around the target of 1.2 mm isotropically [14]. In this study, we randomly selected 200 normal control (NC) subjects and 200 AD patients who were matched in age and gender from the available data according to the information of clinical diagnosis from ADNI. Their MRI data were used to compare the performance of the two versions of AccuBrain in diagnostic accuracy of AD versus NC.

#### EADC-ADNI

EADC-ADNI (http://www.hippocampal-protocol.net/SOPs/index.php) is a globally harmonized protocol (HarP) for manual hippocampal segmentation based on magnetic resonance, which was developed by a task force from European Alzheimer’s Disease Consortium (EADC) and ADNI [15]. This project included 45 normal controls, 45 mild cognitive impairment (MCI) patients and 45 AD patients, and their MRI data and manual hippocampal segmentations [16] were all used in this study. The MRI scans of these subjects were acquired from a mix of 1.5-T and 3.0-T clinical MRI scanners (using the MPRAGE or IR-SPGR technique) following the ADNI scanning protocol which are described elsewhere (http://adni.loni.usc.edu/methods/documents/mri-protocols/) [14].

#### MIRIAD

MIRIAD (http://miriad.drc.ion.ucl.ac.uk/) is a longitudinal dataset that was designed to investigate the feasibility of using MRI as an outcome measure for clinical trials in AD treatments [17]. Participants were scanned at intervals from 2 weeks to 2 years. All images were acquired on a single 1.5T scanner (GE) and the T1-weighted images were acquired using an IR-FSPGR sequence with a resolution of 0.9 mm × 0.9 mm × 1.5 mm. Here, we used the data of the first scans of the baseline, 2 week and 6 week follow-ups for the analyses of intra-scanner reproducibility (n = 62, including 20 NC and 42 AD).

The clinical data of the involved datasets, i.e., (1) EADC-ADNI for evaluation of segmentation accuracy of hippocampus, (2) ADNI for assessing diagnostic accuracy of AD, (3) MIRIAD for intra-scanner reproducibility, are summarized in Table 1.

**Table 1:** Characteristics and MRI measures of the used datasets

Involved analyses	Dataset	Age (years [range])	Female (n [%])	MMSE (range)
Segmentation accuracy of hippocampus	EADC-ADNI			
	*NC* (n = 45)	76 (61–90)	22 (48.9)	29 (27–30)
	*MCI* (n = 45)	75 (60–87)	19 (42.2)	27 (24–30)
	*AD* (n = 45)	74 (63–90)	24 (53.3)	23 (19–26)
Diagnostic accuracy of AD	ADNI			
	*NC* (n = 200)	75 (61–89)	88 (44.0)	29 (24–30)
	*AD* (n = 200)	75 (59–90)	83 (41.5)	23 (19–27)
Intra-scanner reproducibility	MIRIAD			
	*NC* (n = 20)	69 (58–86)	10 (47.6)	29 (27–30)
	*AD* (n = 42)	69 (56–86)	26 (60.5)	20 (13–26)

All the methods involved in this study were carried out in accordance with the Declaration of Helsinki

### Image processing

Regarding the EADC-ADNI dataset where ground truths of hippocampal segmentations were provided, the hippocampal labels were segmented manually once each by five qualified master tracers using standardized HarP guidelines for anatomical landmarks of the hippocampus (http://www.hippocampal-protocol.net/SOPs/LINK_PAGE/FINAL_RELEASE/02_Appendix-II_HarP-UserManual.pdf) [16]. In detail, contours of the hippocampus were manually delineated using MultiTracer 1.0 (https://www.loni.usc.edu/research/software?name=MultiTracer) and the interior of the contour of each coronal slice was then filled using a custom Matlab routine to generate 3D labels of hippocampus [16].

Automated brain volumetry analyses were performed for all the included subjects from the three publicly available third-party datasets with AccuBrain® on T1-weighted [T1W] MRI scans. In detail, given the T1W MRI data, several brain structures and three major brain tissues are segmented automatically based on prior anatomical knowledge specified by experienced radiologists. The anatomical information is automatically transformed into the individual brain. The absolute volumes were calculated from the segmentations of the specific brain structures and the relative volumes were calculated as the ratios of the absolute volumes by the intracranial volume (ICV) of the subject (% of ICV). A quantitative measure that follows the manner of visual rating scale of medial temporal lobe atrophy (i.e., QMTA) was also generated by AccuBrain, which is calculated by the ratio of inferior lateral ventricle (ILV) volume to hippocampal volume [12]. The lobar atrophy index generated by AccuBrain was defined by the ratio of cerebrospinal fluid (CSF) volume within a specific lobar region to the brain parenchyma volume within this lobar region [5, 18].The AD resemblance atrophy index (AD-RAI, ranging from 0 to 1), which indicates the similarity of the brain atrophy pattern with AD, was also generated by AccuBrain [11]. A higher AD-RAI of an individual indicates greater similarity to the brain atrophy pattern in AD patients. This composite MRI-based index was derived from the support vector machine model implemented in AccuBrain based on an in-house training database [11], with the brain volumetric measures quantified by AccuBrain as the predictors.

The brain volumetric measures as mentioned above were quantified with both AccuBrain IV1.2 and AccuBrain IV2.0, where the latest version incorporates updates in brain segmentation algorithm (indicating potential differences in brain volumetric measures between the two versions) while maintaining the same machine learning model for the calculation of AD-RAI.

### Statistical analysis

#### Comparison of segmentation accuracy of hippocampus with EADC-ADNI data

The spatial similarity between automatic hippocampal segmentation of AccuBrain (IV1.2 or IV2.0) and manual segmentation was evaluated with Dice similarity coefficient (DSC), which is calculated as twice the volume of intersection divided by the volume of the union. Numerical precision was measured with intraclass correlation coefficient (ICC) for a single rater using a two-way model for consistency, as well as Pearson’s correlation. Bland–Altman plots were generated for the two versions of AccuBrain respectively to illustrate the differences between AccuBrain and manual hippocampal segmentation. To investigate the significance of difference of the two versions of AccuBrain in hippocampal segmentation, paired sample t-tests were also performed to compare the DSC of segmentation of left and right hippocampus between AccuBrain IV1.2 and AccuBrain IV2.0 with manual segmentations as the reference method. This comparison was not only performed for the entire cohort, but also performed for the sub-cohorts as defined by the diagnosis results (NC, MCI and AD), field strength (1.5T and 3.0T) and manufacturers of the MR scanner (GE, Philips and Siemens).

#### Comparison of diagnosis accuracy for dementia using ADNI data

The receiver operating characteristic curve (ROC) analyses were performed to evaluate the performance of AD-RAI from AccuBrain IV1.2 and AccuBrain IV2.0 for differential diagnosis of AD (n = 200) versus NC (n = 200) using the ADNI data. The performances of the AD-RAI from different versions of AccuBrain were compared with DeLong test with respect to their area under the curves (AUCs) [19]. The default cutoff (i.e., AD-RAI > 0.5 for AD) was applied to estimate the accuracy, sensitivity and specificity of the diagnosis for both versions.

#### Comparison of reproducibility of brain volumetric measures using MIRIAD data

ICC values based on a two-way mixed effects model were calculated to test intra-scanner reproducibility for the brain volumetric measures of AccuBrain in different versions. In detail, we explored the intra-scanner reproducibility of the automated brain volumetry for the scans with short-term intervals (baseline, 2 week and 6 week). These analyses were performed for absolute volumes, relative volumetric measures and AD-RAI, and for both NC and AD groups. The difference of ICC values between IV1.2 and IV2.0 for a specific brain volumetric measure was treated as significant if the point estimate of ICC in one version did not lie in the 95% confidence interval (CI) as mentioned in a previous study [20]. Considering the large number of volumetric measures explored for comparisons for both absolute and relative volumes, we further constrained this criteria of significance with the requirement that the point estimate of ICC in one version should be larger than the upper limit of 95% CI of ICC in the other version by ≥ 0.005.

## Results

### Comparison of segmentation accuracy of hippocampus

**Fig. 1:**
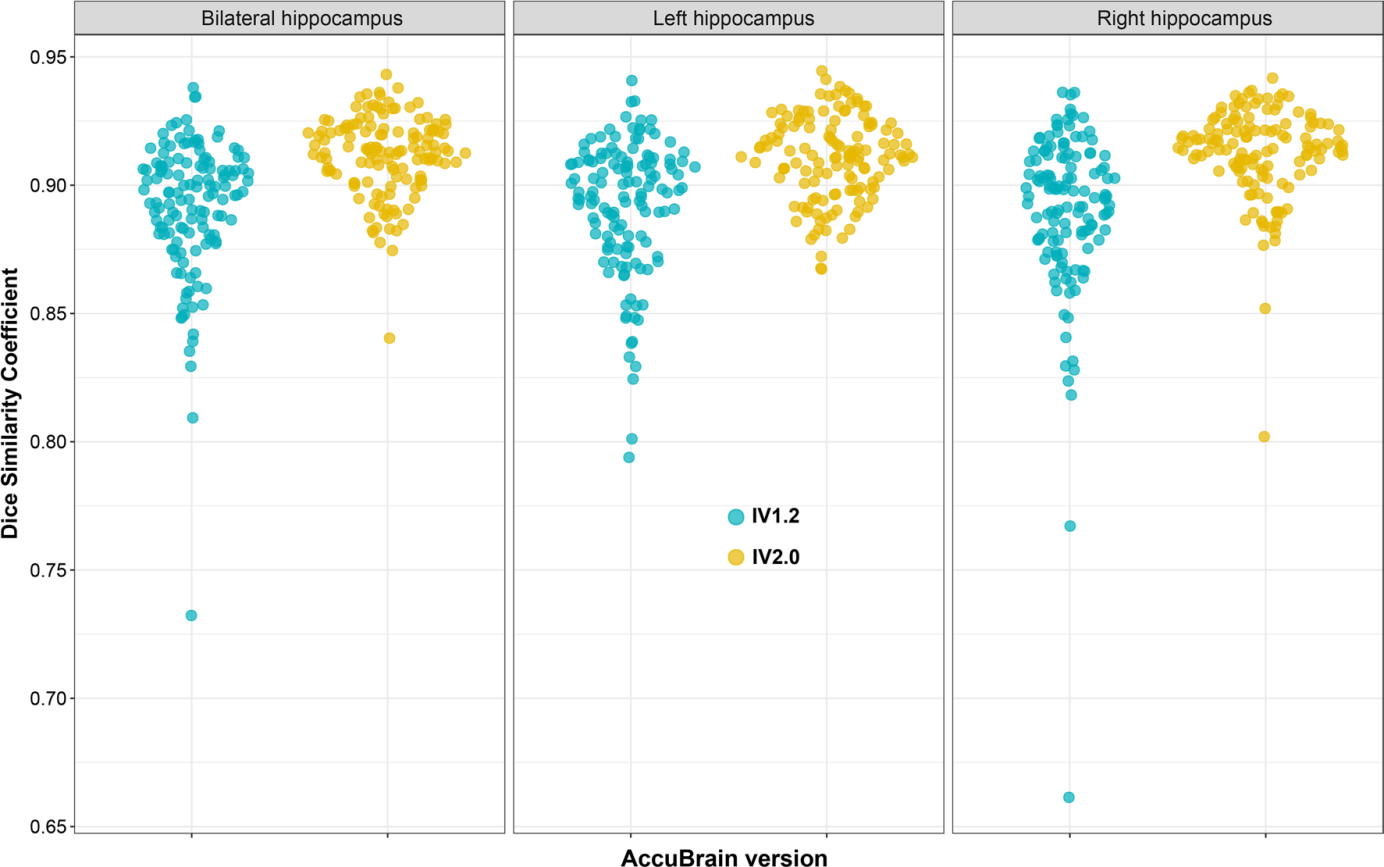
Distribution of dice similarity coefficients of hippocampal segmentations

**Fig. 2:**
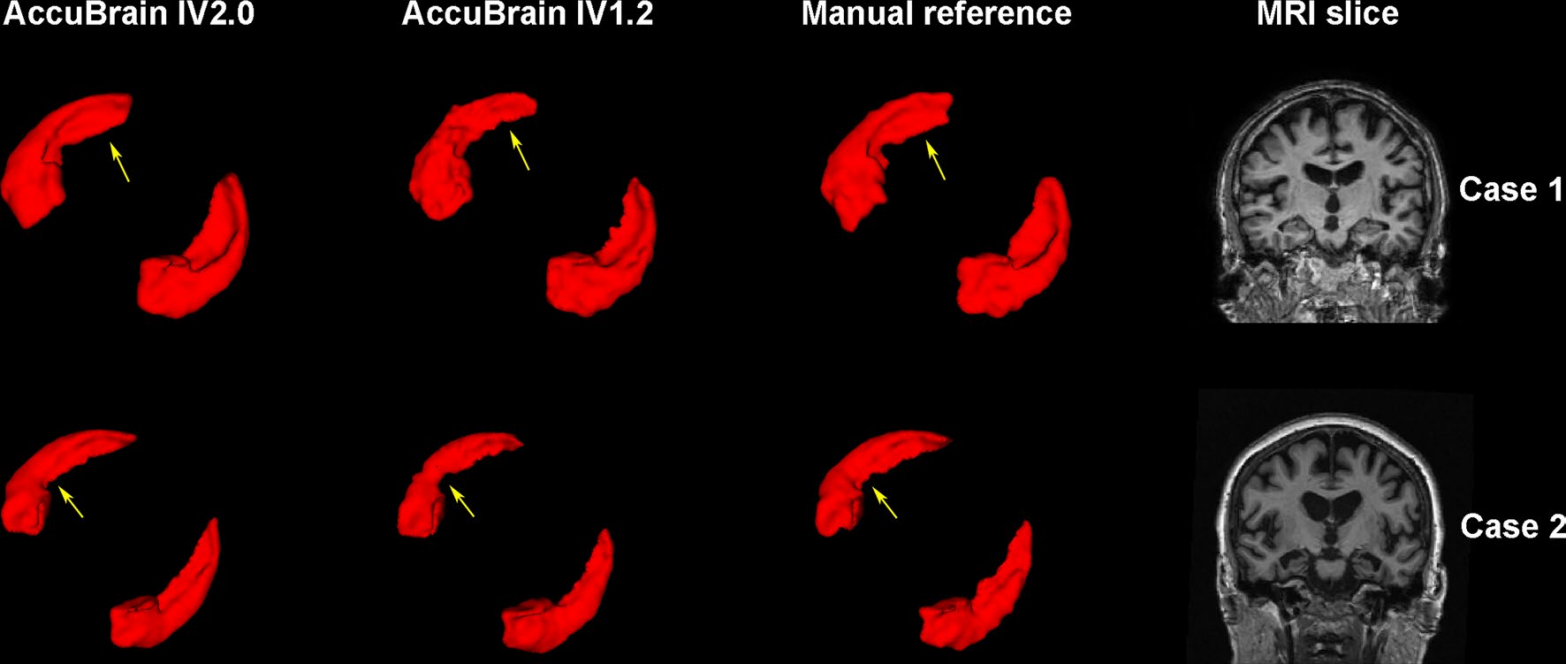
Sample hippocampal segmentation results of AccuBrain IV2.0, AccuBrain IV1.2 compared with manual reference together with original MRI slices. Hippocampal segmentations for Case 1 (aged 82 years, male, with normal cognition): DSC of 0.913 for AccuBrain IV2.0 and 0.894 for AccuBrain IV1.2; Hippocampal segmentations for Case 2 (aged 72 years, female, diagnosed with AD): DSC of 0.902 for AccuBrain IV2.0 and 0.849 for AccuBrain IV1.2. The displayed DSC values refer to accuracy of total hippocampal segmentation. The yellow arrows point to typical areas where the segmentation of AccuBrain IV2.0 is more consistent with manual reference than that of AccuBrain IV1.2

**Fig. 3:**
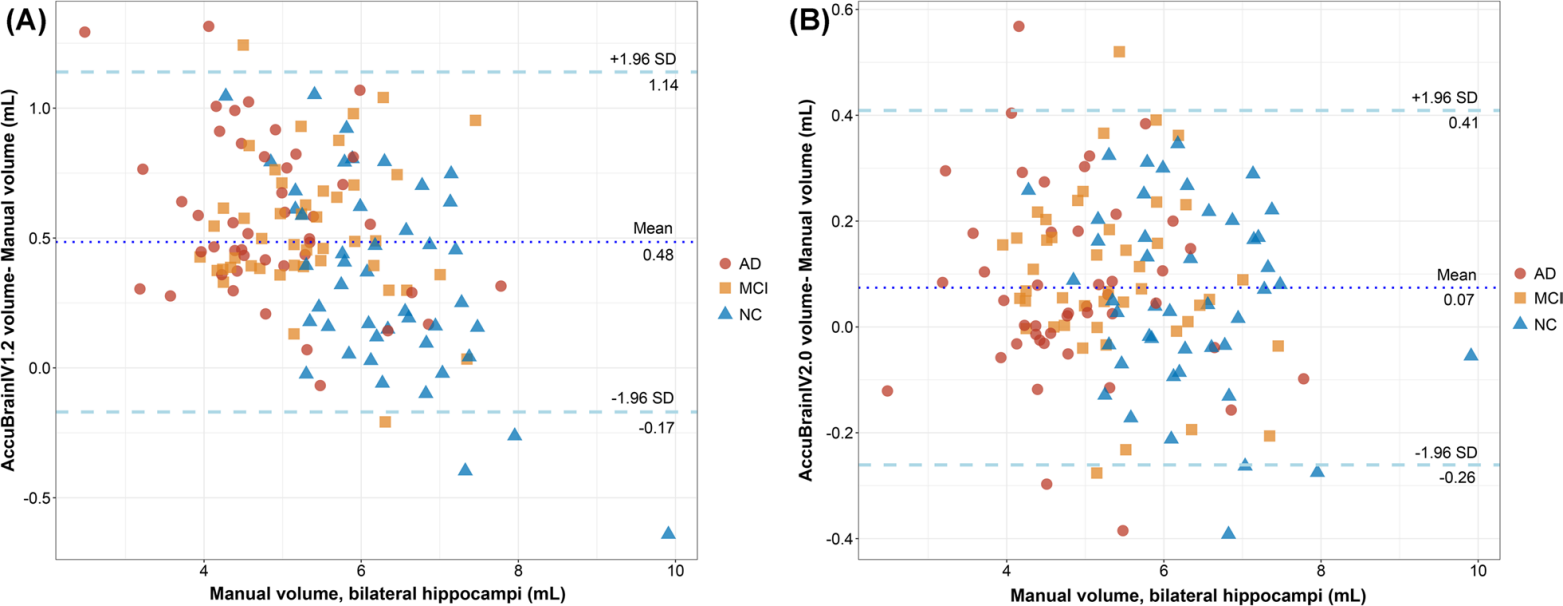
Bland–Altman plots for hippocampal volumes of AccuBrain IV1.2 (**A**) and AccuBrain IV2.0 (**B**) compared with those of manual segmentation. *NC* normal cohort, *MCI* mild cognitive impairment, *AD* Alzheimer’s disease

In the comparison of the two versions of AccuBrain for hippocampal segmentation with manual segmentation as the reference, IV2.0 presented significantly higher DSC than IV1.2 (Table 2) either in the entire cohort (IV2.0 vs. IV1.2: 0.910 vs. 0.892 [*p* < 0.001] for left hippocampus, 0.912 vs. 0.890 [*p* < 0.001] for right hippocampus, Table 2; Fig. 1) or in the subgroups (all with *p* < 0.001) defined by diagnosis, field strength or manufacturer (Table 2). Representative hippocampal segmentation results were also shown in Fig. 2 to illustrate the superior performance of IV2.0 over IV1.2 regarding volume overlap. In terms of numeric precision, hippocampal volumes of AccuBrain IV2.0 presented stronger correlation with manual segmentation than that of AccuBrain IV1.2, with ICC of 0.989 (95% CI 0.984–0.992, *p* < 0.001) versus 0.955 (95% CI 0.938–0.968, *p* < 0.001) for the left hippocampus and 0.983 (95% CI 0.976–0.988) versus 0.941 (95% CI 0.918–0.957) for the right hippocampus. AccuBrain IV2.0 also presented higher Pearson’s r values for the hippocampal volumes than AccuBrain IV1.2 (0.989 vs. 0.958 for left hippocampus and 0.983 vs. 0.947 for right hippocampus). Bland–Altman plots of absolute differences between AccuBrain and manual segmentation volumes (Fig. 3) showed general slight volume overestimation by AccuBrain IV1.2 (means of + 0.48 mL for bilateral hippocampus), and AccuBrain IV2.0 presented even smaller overestimation (means of + 0.07 mL) while maintaining a similar ratio of outliers and smaller SD of volumetric bias compared with AccuBrain IV1.2 (0.33 mL vs. 0.65 mL).

**Table 2:** Mean DSC (SD) of AccuBrain IV2.0 and AccuBrain IV1.2 with manual hippocampal segmentation as reference method

	Left hippocampus			Right hippocampus		
	AccuBrain IV2.0	AccuBrain IV1.2	*p*	AccuBrain IV2.0	AccuBrain IV1.2	*p*
All (n = 135)	0.910 (0.016)	0.892 (0.026)	< 0.001	0.912 (0.018)	0.890 (0.033)	< 0.001
Diagnosis						
NC (n = 45)	0.919 (0.015)	0.907 (0.019)	< 0.001	0.921 (0.012)	0.904 (0.018)	< 0.001
MCI (n = 45)	0.910 (0.013)	0.889 (0.020)	< 0.001	0.913 (0.015)	0.889 (0.029)	< 0.001
AD (n = 45)	0.902 (0.016)	0.880 (0.030)	< 0.001	0.903 (0.021)	0.877 (0.041)	< 0.001
Field strength						
1.5T (n = 68)	0.916 (0.014)	0.894 (0.026)	< 0.001	0.913 (0.019)	0.890 (0.039)	< 0.001
3.0T (n = 67)	0.905 (0.017)	0.890 (0.026)	< 0.001	0.911 (0.016)	0.890 (0.025)	< 0.001
Manufacturer						
GE (n = 45)	0.912 (0.014)	0.894 (0.026)	< 0.001	0.916 (0.013)	0.900 (0.023)	< 0.001
Philips (n = 44)	0.914 (0.015)	0.893 (0.023)	< 0.001	0.916 (0.016)	0.888 (0.028)	< 0.001
S (n = 46)	0.905 (0.018)	0.889 (0.028)	< 0.001	0.905 (0.021)	0.882 (0.042)	< 0.001

The significance levels (p) of the paired sample tests for the entire cohort and specific sub-cohorts are displayed

*DSC* Dice similarity coefficient, *NC* normal control, *MCI* mild cognitive impairment, *AD* Alzheimer’s disease

### Comparison of diagnosis accuracy for dementia with ADNI data

**Fig. 4:**
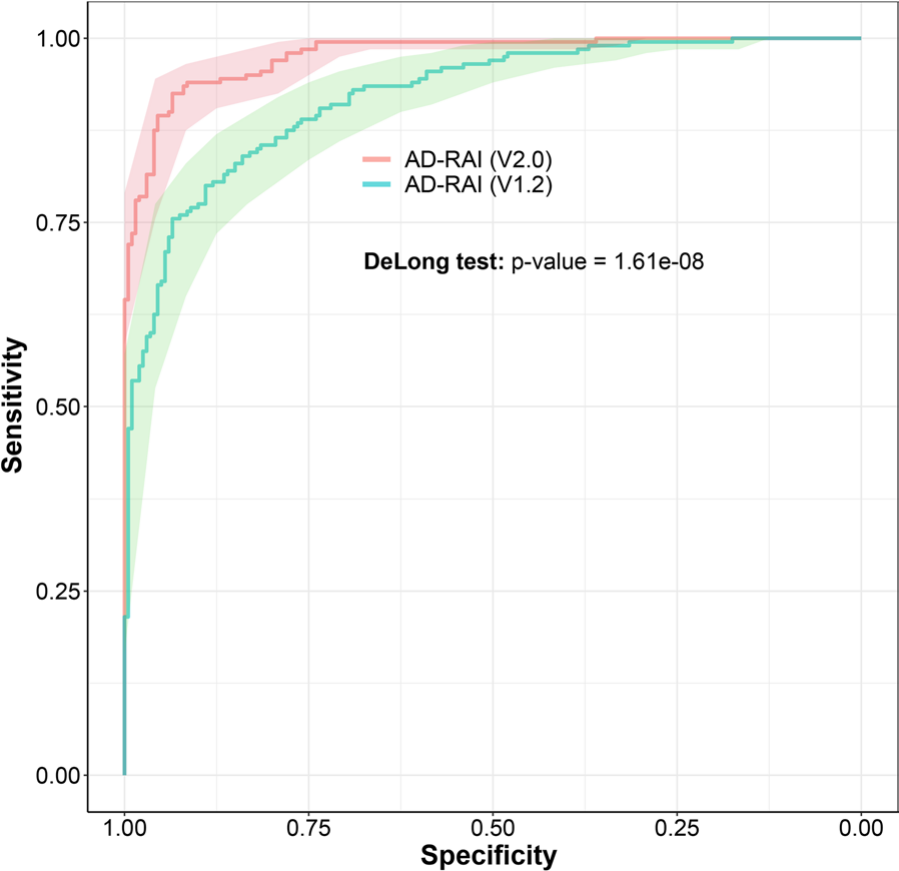
ROC curves of AD-RAI for differentiation of AD from NC (shaded areas represent 95% confidence intervals for the ROC curves). The AUC of AD-RAI from AccuBrain IV2.0 was significantly higher than that from AccuBrain IV1.2 (the *p* value = 1.61e−08 for the comparison of the ROC curves with DeLong test)

In the analyses of diagnosis of AD, AccuBrain IV2.0 performed statistically better than IV1.2 (AUC: 0.977 vs. 0.921, *p* < 0.001 with DeLong test). This difference was also visualized in Fig. 4 as the ribbons of 95% CI (i.e., shaded areas) of their ROC curves rarely overlapped. With the default cutoff (AD-RAI > 0.5 for AD), AccuBrain IV2.0 also presented higher accuracy (90.5% vs. 83.5%), sensitivity (94.0% vs. 84.5%) and specificity (87.0% vs. 82.5%) than IV1.2.

### Comparison of reproducibility of brain volumetric measures using MIRIAD data

Regarding the analyses of intra-scanner reproducibility with short-term intervals, the point estimates of ICC values were all larger than 0.95 for absolute volumes and AD-RAI (Table 3) and generally larger than 0.90 for relative volumetric measures (Table 4). These two versions generally presented no significant differences for the ICC values for most brain volumetric measures in NC or AD. Nonetheless, IV2.0 presented significantly higher ICC than IV1.2 for brain parenchyma (relative volume), bilateral hippocampus (both absolute and relative volumes), right frontal lobe atrophy, left occipital lobe atrophy, right temporal lobe atrophy, and bilateral parietal lobe atrophy in NC and for right hippocampus (both absolute and relative volumes) in AD, while IV1.2 presented higher ICC than IV2.0 for gray matter (relative volume), right amygdala (both absolute and relative volumes), bilateral temporal lobe atrophy and right insular atrophy in AD.

**Table 3:** ICC for intra-scanner reproducibility regarding absolute volumes and AD-RAI

Absolute volumes	NC (n = 20)		AD (n = 42)	
	AccuBrain IV2.0	AccuBrain IV1.2	AccuBrain IV2.0	AccuBrain IV1.2
Intracranial volume	0.990 (0.979, 0.996)	0.995 (0.988, 0.998)	0.990 (0.983, 0.994)	0.996 (0.993, 0.997)
Brain parenchyma	0.99 (0.98, 1.00)	0.98 (0.96, 0.99)	0.99 (0.98, 1.00)	0.99 (0.98, 1.00)
White matter	0.97 (0.95, 0.99)	0.96 (0.92, 0.98)	0.97 (0.95, 0.98)	0.96 (0.94, 0.98)
Gray matter	0.99 (0.97, 0.99)	0.98 (0.96, 0.99)	0.97 (0.95, 0.98)	0.98 (0.97, 0.99)
Hippocampus L	0.98 (0.96, 0.99)*	0.91 (0.83, 0.96)	0.99 (0.99, 1.00)	0.99 (0.98, 0.99)
Hippocampus R	0.99 (0.97, 0.99)*	0.90 (0.80, 0.95)	0.992 (0.987, 0.996)*	0.977 (0.961, 0.987)
Amygdala L	0.95 (0.90, 0.98)	0.94 (0.88, 0.97)	0.98 (0.96, 0.99)	0.98 (0.97, 0.99)
Amygdala R	0.93 (0.87, 0.97)	0.94 (0.88, 0.97)	0.976 (0.961, 0.986)	0.991 (0.984, 0.995)*
Lateral ventricle L	0.999 (0.999, 1.000)	0.999 (0.998, 1.000)	0.999 (0.998, 0.999)	0.999 (0.999, 0.999)
Lateral ventricle R	0.999 (0.997, 0.999)	0.997 (0.995, 0.999)	0.999 (0.998, 0.999)	0.999 (0.998, 0.999)
Inf Lat Vent L	0.991 (0.981, 0.996)	0.976 (0.951, 0.990)	0.995 (0.992, 0.997)	0.995 (0.992, 0.997)
Inf Lat Vent R	0.976 (0.951, 0.990)	0.985 (0.969, 0.994)	0.993 (0.988, 0.996)	0.995 (0.991, 0.997)
Thalamus L	0.98 (0.96, 0.99)	0.98 (0.95, 0.99)	0.98 (0.97, 0.99)	0.98 (0.97, 0.99)
Thalamus R	0.98 (0.97, 0.99)	0.98 (0.96, 0.99)	0.98 (0.97, 0.99)	0.98 (0.97, 0.99)
Caudate L	0.99 (0.97, 0.99)	0.99 (0.98, 1.00)	0.99 (0.98, 0.99)	0.98 (0.97, 0.99)
Caudate R	0.99 (0.98, 1.00)	0.99 (0.98, 1.00)	0.98 (0.97, 0.99)	0.99 (0.98, 0.99)
Putamen L	0.98 (0.96, 0.99)	0.99 (0.97, 0.99)	0.98 (0.97, 0.99)	0.98 (0.97, 0.99)
Putamen R	0.96 (0.93, 0.99)	0.96 (0.92, 0.98)	0.97 (0.95, 0.98)	0.97 (0.96, 0.98)
Pallidum L	0.96 (0.92, 0.98)	0.95 (0.90, 0.98)	0.96 (0.93, 0.98)	0.97 (0.94, 0.98)
Pallidum R	0.95 (0.91, 0.98)	0.96 (0.92, 0.98)	0.98 (0.97, 0.99)	0.97 (0.95, 0.98)
Midbrain	0.98 (0.96, 0.99)	0.97 (0.94, 0.99)	0.99 (0.99, 1.00)	0.99 (0.99, 1.00)
Pons	0.99 (0.98, 1.00)	0.99 (0.98, 1.00)	0.995 (0.991, 0.997)	0.996 (0.993, 0.998)
Cerebellum	0.99 (0.98, 1.00)	0.99 (0.98, 1.00)	0.99 (0.99, 1.00)	0.99 (0.99, 1.00)
AD-RAI	0.983 (0.965,0.993)	0.984 (0.967,0.993)	0.982 (0.970, 0.990)	0.979 (0.965, 0.988)

The ICC values were displayed in terms of point estimate and 95% CI with lower and upper limits

*Significantly different based on point estimate of AccuBrain IV2.0 not lying within the confidence interval (CI) of AccuBrain IV1.2, where the point estimate of ICC for this version (labeled with *) is larger than the upper limit of 95% CI of the other version by ≥ 0.005

**Table 4:** ICC for intra-scanner reproducibility regarding relative volumes

Relative volumes	NC (n = 20)		AD (n = 42)	
	AccuBrain IV2.0	AccuBrain IV1.2	AccuBrain IV2.0	AccuBrain IV1.2
Brain parenchyma	0.97 (0.93, 0.99)*	0.91 (0.83, 0.96)	0.86 (0.78, 0.92)	0.87 (0.80, 0.92)
White matter	0.95 (0.89, 0.98)	0.90 (0.80, 0.95)	0.86 (0.78, 0.92)	0.79 (0.68, 0.87)
Gray matter	0.96 (0.93, 0.98)	0.93 (0.86, 0.97)	0.75 (0.63, 0.85)	0.87 (0.80, 0.93)*
Hippocampus L	0.98 (0.97, 0.99)*	0.89 (0.78, 0.95)	0.99 (0.98, 0.99)	0.98 (0.97, 0.99)
Hippocampus R	0.98 (0.97, 0.99)*	0.89 (0.78, 0.95)	0.99 (0.99, 1.00)*	0.97 (0.96, 0.98)
Amygdala L	0.94 (0.88, 0.97)	0.92 (0.84, 0.96)	0.97 (0.95, 0.98)	0.98 (0.97, 0.99)
Amygdala R	0.91 (0.83, 0.96)	0.95 (0.89, 0.98)	0.96 (0.93, 0.98)	0.99 (0.98, 0.99)*
Lateral ventricle L	0.999 (0.998, 1.000)	0.999 (0.997, 0.999)	0.999 (0.998, 0.999)	0.999 (0.998, 0.999)
Lateral ventricle R	0.998 (0.996, 0.999)	0.997 (0.993, 0.999)	0.999 (0.998, 0.999)	0.999 (0.998, 0.999)
Inf Lat Vent L	0.989 (0.978, 0.995)	0.973 (0.944, 0.988)	0.995 (0.992, 0.997)	0.995 (0.992, 0.997)
Inf Lat Vent R	0.973 (0.945, 0.988)	0.986 (0.970, 0.994)	0.992 (0.987, 0.996)	0.995 (0.991, 0.997)
Thalamus L	0.98 (0.96, 0.99)	0.97 (0.93, 0.99)	0.97 (0.96, 0.98)	0.98 (0.97, 0.99)
Thalamus R	0.99 (0.97, 0.99)	0.98 (0.96, 0.99)	0.98 (0.96, 0.99)	0.98 (0.97, 0.99)
Caudate L	0.99 (0.98, 1.00)	0.99 (0.98, 1.00)	0.98 (0.97, 0.99)	0.97 (0.95, 0.98)
Caudate R	0.99 (0.97, 0.99)	0.99 (0.98, 1.00)	0.976 (0.961, 0.986)	0.987 (0.979, 0.993)
Putamen L	0.99 (0.98, 1.00)	0.99 (0.99, 1.00)	0.98 (0.97, 0.99)	0.98 (0.97, 0.99)
Putamen R	0.98 (0.96, 0.99)	0.98 (0.96, 0.99)	0.97 (0.95, 0.98)	0.97 (0.96, 0.99)
Pallidum L	0.98 (0.95, 0.99)	0.96 (0.91, 0.98)	0.94 (0.90, 0.96)	0.95 (0.91, 0.97)
Pallidum R	0.97 (0.94, 0.99)	0.97 (0.93, 0.99)	0.97 (0.94, 0.98)	0.95 (0.92, 0.97)
Midbrain	0.98 (0.95, 0.99)	0.97 (0.94, 0.99)	0.979 (0.966, 0.988)	0.989 (0.982, 0.994)
Pons	0.99 (0.98, 1.00)	0.99 (0.99, 1.00)	0.99 (0.99, 1.00)	0.99 (0.99, 1.00)
Cerebellum	0.991 (0.981, 0.996)	0.997 (0.993, 0.999)	0.983 (0.972, 0.990)	0.991 (0.985, 0.995)
Frontal lobe atrophy L	0.948 (0.894, 0.977)	0.919 (0.839, 0.964)	0.940 (0.903, 0.965)	0.921 (0.873, 0.954)
Frontal lobe atrophy R	0.970 (0.939, 0.987)*	0.917 (0.835, 0.963)	0.924 (0.878, 0.956)	0.918 (0.868, 0.952)
Occipital lobe atrophy L	0.958 (0.915, 0.982)*	0.892 (0.790, 0.952)	0.932 (0.890, 0.960)	0.947 (0.914, 0.969)
Occipital lobe atrophy R	0.910 (0.823, 0.960)	0.868 (0.747, 0.941)	0.875 (0.802, 0.926)	0.928 (0.884, 0.958)
Temporal lobe atrophy L	0.970 (0.939, 0.987)	0.934 (0.868, 0.971)	0.918 (0.869, 0.952)	0.965 (0.942, 0.980)*
Temporal lobe atrophy R	0.976 (0.951, 0.990)*	0.876 (0.760, 0.944)	0.914 (0.862, 0.950)	0.966 (0.944, 0.980)*
Parietal lobe atrophy L	0.972 (0.941, 0.988)*	0.918 (0.837, 0.964)	0.968 (0.947, 0.981)	0.977 (0.962, 0.987)
Parietal lobe atrophy R	0.975 (0.948, 0.989)*	0.907 (0.817, 0.959)	0.973 (0.955, 0.984)	0.973 (0.955, 0.984)
Cingulate lobe atrophy L	0.976 (0.949, 0.989)	0.971 (0.939, 0.987)	0.949 (0.916, 0.970)	0.966 (0.943, 0.980)
Cingulate lobe atrophy R	0.980 (0.959, 0.992)	0.968 (0.935, 0.986)	0.948 (0.915, 0.970)	0.948 (0.915, 0.970)
Insular atrophy L	0.985 (0.969, 0.994)	0.970 (0.939, 0.987)	0.899 (0.838, 0.940)	0.942 (0.905, 0.966)
Insular atrophy R	0.972 (0.942, 0.988)	0.960 (0.918, 0.983)	0.945 (0.910, 0.968)	0.981 (0.969, 0.989)*
Cerebellum atrophy	0.943 (0.885, 0.975)	0.901 (0.806, 0.956)	0.930 (0.886, 0.959)	0.921 (0.872, 0.954)
QMTA L	0.988 (0.976, 0.995)	0.969 (0.937, 0.987)	0.995 (0.991, 0.997)	0.994 (0.990, 0.997)
QMTA R	0.98 (0.95, 0.99)	0.98 (0.96, 0.99)	0.99 (0.99, 1.00)	0.99 (0.99, 1.00)

The ICC values were displayed in terms of point estimate and 95% CI with lower and upper limits

*Significantly different based on point estimate of AccuBrain IV2.0 not lying within the confidence interval (CI) of AccuBrain IV1.2, where the point estimate of ICC for this version (labeled with *) is larger than the upper limit of 95% CI of the other version by ≥ 0.005

## Discussion

In this study, we investigated the influence of version update of AccuBrain (i.e., IV2.0 vs. IV1.2) in segmentation accuracy, intra-scanner reproducibility, and diagnostic accuracy of AD, which provided suggestions of AccuBrain for future use.

Regarding the segmentation accuracy, we compared AccuBrain IV2.0 and AccuBrain IV1.2 for hippocampal segmentation with manual segmentation as the reference standard, which was partly involved in a previous study which focused on the comparison of AccuBrain (IV1.2) with FreeSurfer using the same dataset [2]. Here, we found that IV2.0 presented significant increase of spatial accuracy (DSC) compared with IV1.2 for both left and right hippocampus and for both the entire cohort and the subgroups defined by diagnosis, field strength of MR scans or manufacturer of MR scanners (Table 2). This kind of improvements from the version update were also observed for numeric precision (ICC and Pearson correlation), and absolute volumetric differences (less bias from manual results as shown in Bland-Altman plots, Fig. 3). Considering the better performance of AccuBrain IV1.2 than FreeSurfer in hippocampal segmentation as reported in the previous study [2], the superiority of AccuBrain over FreeSurfer would be further increased with the updates in AccuBrain IV2.0.

Regarding the diagnostic accuracy of AD with AD-RAI, AccuBrain IV2.0 presented significantly higher AUC than AccuBrain IV1.2 (0.977 vs. 0.921, Fig. 4). Here, the AUC of AD-RAI in AccuBrain IV1.2 (0.921) was in line with a previous study where the older version of AccuBrain (IV1.2) was also used for clinical diagnosis of AD vs. NC in another dataset (AUC = 92%) [12]. As AD-RAI has also been used to identify early stages of AD with the older version of AccuBrain (IV1.2) [11], the improvement of diagnostic accuracy of AD for AD-RAI in IV2.0 may indicate its better performance for early detection of AD as AD-RAI depicts the similarity with AD-like brain atrophy pattern, although further validations are needed. Of note, the update of AccuBrain only involves improvements of segmentation algorithms but not the machine learning model that generates AD-RAI. The improvement of accuracy of AD-RAI in differential diagnosis (AD vs. NC) should result from the improved segmentation accuracy of the brain structures (e.g., hippocampus as indicated from this study). In fact, AD-RAI is generated by a machine learning model that includes the brain volumetric measures that are involved in AD-like brain atrophy, such as hippocampus and ILV [10], where the improvement of hippocampal segmentation accuracy has been demonstrated directly (Figs. 1, 2 and 3). In this regard, the previous studies that applied brain volumetric measures of AccuBrain IV1.2 for differential diagnosis (e.g., vascular dementia vs. AD [9]) with machine learning models may also achieve better performance with the version update, while further validations are needed.

Regarding the analyses of intra-scanner reproducibility for scans with short-term intervals, AccuBrain IV2.0 and AccuBrain IV1.2 generally showed similar ICC for most of the brain volumetric measures. Among the several brain volumetric measures where the two versions presented significant differences in reproducibility, the results also did not favor one version over the other for either NC or AD population. In detail, IV2.0 presented higher ICCs in more volumetric measures than IV1.2 (especially in NC group) for reproducibility of scans with short-term intervals (Tables 3 and 4). As most of the ICC values of both versions for either analysis of intra-scanner reproducibility were > 0.90 and that the largest difference between point estimates of ICC for the two versions was < 0.10, both IV1.2 and IV2.0 should be feasible for use in research or clinical settings with repetitive or follow-up scans on the same scanner.

There are several limitations that should be considered in this study. Firstly, the databases involved in this study were identified based on the cognitive status (i.e., NC and AD), and no other population, for example, those with lesions in the brain such as stroke, were enrolled for analysis. Although the investigations in this study may demonstrate the feasibility of both versions of AccuBrain for automated brain volumetry in populations with various cognitive status, further validations are needed in patients with other neurological diseases. In addition, although we demonstrated that both versions presented good and comparable intra-scanner reproducibility, inter-scanner reproducibility was not tested due to the lack of available data. Further validations are needed to compare the two versions of AccuBrain regarding their volumetric measures quantified on different scanners (e.g., with different manufacturer or field strength).

In conclusion, AccuBrain IV2.0 performed significantly better than IV1.2 in hippocampal segmentation and diagnostic accuracy of AD vs. NC, while both versions should be feasible for use as the magnitude of difference between their performances was not large. In addition, our findings suggest no significant differences between versions or favor one version over the other regarding the intra-scanner reproducibility from different MRI scans on the same scanner.

## Data Availability

The data that support the findings of this study are available from EADC-ADNI, ADNI and MIRIAD but restrictions apply to the availability of these data, which were used under license for the current study. Therefore, the data would be available from the authors upon reasonable request and with permission of the aforementioned organizations.

## Abbreviations

AD: Alzheimer’s disease
ADNI: Alzheimer’s disease neuroimaging initiative
AD-RAI: AD resemblance atrophy index
AUC: Area under the curve
CI: Confidence interval
CSF: Cerebrospinal fluid
DSC: Dice similarity coefficient
EADC: European Alzheimer’s disease consortium
ICC: Intraclass correlation coefficient
ICV: Intracranial volume
ILV: Inferior lateral ventricle
IR-FSPGR: Inversion recovery prepared fast spoiled gradient recalled
MCI: Mild cognitive impairment
MPRAGE: Magnetization-prepared rapid gradient echo
MRI: Magnetic resonance imaging
NC: Normal control
QMTA: Quantitative measure of medial temporal lobe atrophy
ROC: Receiver operating characteristic curve
T1W: T1-weighted
TLE: Temporal lobe epilepsy
